# Evaluation of Antioxidant Compounds and Total Sugar Content in a Nectarine [*Prunus persica* (L.) Batsch] Progeny

**DOI:** 10.3390/ijms12106919

**Published:** 2011-10-19

**Authors:** Walid Abidi, Sergio Jiménez, María Ángeles Moreno, Yolanda Gogorcena

**Affiliations:** Departamento de Pomología, Estación Experimental de Aula Dei (CSIC), Apartado 13034, Zaragoza 50080, Spain; E-Mails: wabidi@eead.csic.es (W.A.); sjimenez@eead.csic.es (S.J.); mmoreno@eead.csic.es (M.A.M.)

**Keywords:** antioxidant capacity, flavonoids, total phenolics, vitamin C

## Abstract

Epidemiological studies suggest that consumption of fruit rich in phenolic compounds is associated with health-protective effects due to their antioxidant properties. For these reasons quality evaluation has become an important issue in fruit industry and in breeding programs. Phytochemical traits such as total phenolics, flavonoids, anthocyanins, L-ascorbic acid, sugar content and relative antioxidant capacity (RAC) were analyzed over four years in flesh fruit of an F1 population “Venus” × “Big Top” nectarines. Other traits such as harvesting date, yield, fruit weight, firmness, soluble solids concentration (SSC), pH, titratable acidity (TA) and ripening index (RI) were also determined in the progeny. Results showed high variability among genotypes for all analyzed traits. Total phenolics and flavonoids showed significant positive correlations with RAC implying that both are important antioxidant bioactive compounds in peaches. We found genotypes with enhanced antioxidant capacity and a better performance than progenitors, and in consequence the best marketability.

## 1. Introduction

Peach [*Prunus persica* (L.) Batsch] production has an important place in the world (18.6 million tons in 2009) with a cultivated area of around 1.6 million ha [1]. Peaches and nectarines are, after apples, the second most important fruit crop in the European Union (EU), with a production of 4.1 million tons in 2009 and a cultivated area of around 245,191 ha [1]. Spain is the third largest producer in the world, after China and Italy, and the second in Europe, with a production of 1.2 million tons in 2009 and a cultivated area of 72,000 ha [1].

Peaches are a popular summer fruit and there has been an increasing interest in their nutritional value [2]. Many epidemiological studies suggest that increased fruit consumption decreases the risk of several degenerative diseases including atherosclerosis, heart and brain disorders, and different types of cancer [3] which are still responsible for the highest mortality rate in Western countries [4]. In particular, the consumption of peaches can suppress reactive oxygen species (ROS) in human plasma and provide protection from chronic diseases [5]. Fruits have recently been accepted as a functional food, because of its low caloric content along with its high level of antioxidant and nutritional compounds, such as vitamins, phenols, minerals or fiber that could play an important role in preventing oxidative stress [6]. The phenolic compounds (anthocyanins, flavonoids, *etc.*) give fruits both desirable qualities like color and antioxidant properties and undesirable qualities like astringency and bitterness [7]. Slimestad *et al.* [8] reported that there is a correlation between taste (astringency, bitterness) and content of phenolic compounds which have an important role in the natural defense mechanisms and health benefits of fruits. Moreover, Koh *et al.* [9] reported that flavonoids are particularly interesting as they are potent *in vitro* antioxidants and are thought to play key roles in many of the processes underlying vascular dysfunction.

In recent years, there has been an increased interest in breeding programs identifying and quantifying phenolic substances in fruit in order to evaluate their potential health-promoting properties, as well as their visual appearance (pigmentation and browning) and taste (astringency) [6,10]. Peaches could be of great interest as an important antioxidant source and intake of these fruits may provide health-promoting advantages [11].

Apart from the antioxidant evaluation, peach breeding programs have stressed the importance of taste in the selection of new cultivars [12]. Orazem *et al*. [6] reported that the edible quality of peaches depends to a great extent on their sweetness, and that the amounts of sucrose, sorbitol and malic acid correlate positively with the taste and aroma of peach fruit. Sweetness and acidity are the most important factors affecting consumer acceptability of stone fruits and these factors are strictly correlated [11]. Sucrose, glucose and fructose are the main sugars in peaches [13,14], and in ripe fruit, these sugars comprise about 60% of the soluble solids concentration (SSC). The relative concentrations of these sugars also influence sweetness, as fructose is 2.3 times and 1.7 times sweeter than glucose and sucrose, respectively [15].

The main objective of this work was to evaluate, in a nectarine segregating F1 population derived from “Venus” × “Big Top” over a 4-year study, the existing phenotypic diversity of antioxidant compounds and total sugar content among genotypes, and to study the relationships among agronomic and biochemical fruit quality traits. The correlations between biochemical and agronomic traits will be very useful in peach breeding programs. The ultimate objective of this study was to select superior genotypes with enhanced antioxidant capacity in fruits that will benefit consumers with health-promoting properties.

## 2. Results and Discussion

### 2.1. Agronomical and Basic Fruit Quality Traits

Traits were evaluated in both parents and each seedling separately over four years (2007–2010) of study (Table 1). Mean values of fruit weight, firmness, soluble solids content (SSC), pH, titratable acidity (TA) and the ripening index ratio (RI = SSC/TA) were calculated for parents (“Venus” and “Big Top”) as well as for the 75 individual seedling progeny. Results showed high variability among genotypes for the different agronomic and fruit quality traits evaluated. Means for all analyzed traits were inside the interval values obtained for the parents and exhibited continuous variation, which is typical of quantitative or polygenic inheritance. The fruit weight varied greatly among genotypes (190.2 ± 3.8 g) as a consequence of the variability in tree production and fruits number for each seedling. Fruit weight is a major quantitative inherited factor determining yield, fruit quality and consumer acceptability [16]. The mean value for yield in the progeny for 2007–2010 was 6.9 ± 0.3 kg per tree with high variability among genotypes, which may occur due to year and genotype (data not shown). These values were similar to those obtained by Cantín *et al.* [17] in the same population during three years. Milatović *et al.* [18] reported that yield of the peach tree depends on different factors, such as density of flower buds and flowers, fruit set, fruit size, winter and late spring freeze damage, precipitation amount, and orchard management. The variation for agronomic and basic biochemical fruit quality traits among years of study showed lower yield for years 2008 and 2010 (5.9 and 5.4 kg, respectively) compared with the mean value of four years (6.9 ± 0.3 kg ) and consequently higher fruit weight for these two years (209.0 and 214.2 g, respectively) than the average weight (190.2 ± 3.8 g). This variability in annual yield was mainly due to rains damage which occurred in the full blooming of the population limiting the number of available fruits. The pH, fruit firmness and SSC showed low variability among years (data not shown).

Regarding flesh fruit firmness, it ranged from 24.2 to 50.7 N with higher variability among seedlings. The two progenitors and some genotypes of the progeny showed firmness values higher than 35 N, which has been defined as the threshold between mature and immature fruit [19]. Our analysis revealed a mean firmness of 39.2 N (4.08 kg/cm^2^) which is lower than the maximum level of fruit firmness for marketing fresh peaches and nectarines, set by the EU at a 6.5 kg/cm^2^ (=63.7 N), using a 8 mm diameter probe (Commission Regulation EC, No.1861/2004 of 28 October 2004).

Regarding SSC, the population showed values from 11.2 to 17.5 °Brix with a mean of 13.9 ± 0.2 °Brix, which is greater than the minimum (8 °Brix) established by the EU to market peaches and nectarines (R-CE No.1861/2004). Kader [20] considered mean values of SSC over 10 °Brix as the minimum value for consumer acceptance for yellow-flesh nectarines, which is the case in our progeny. The variability found in SSC among seedlings can be explained by the quantitative performance of this quality trait [21].

The pH values varied from 3.2 to 4.0 with a mean value of 3.6 ± 0.1, which are values of normal acidity fruits. The progeny showed acid and non-acid fruits, since fruit with a pH higher than 4.0 at maturity are considered as non-acid [22]. The progeny showed variability of TA among genotypes with a mean value of 0.7 ± 0.1 g malic acid per 100 g fresh weight (FW), which is lower than the maximum limit (0.9%) for normal acidity peaches [23]. “Venus” is an acid nectarine (TA = 0.7%), and “Big Top” is a non-acid nectarine (TA = 0.4%), which explains the segregation of this trait in the progeny.

The ripening index (RI = SSC/TA) ranged from 13.8 to 35.8 among genotypes, depending on their SSC and TA values. In peaches, the RI is a major organoleptic quality trait of the mature fruit and is commonly used as a quality index [24]. The relationship between TA and SSC has an important role in consumer acceptance of some apricot, peach, nectarine and plum cultivars. Crisosto *et al.* [25] reported that in the case of cultivars with TA > 0.9% and SSC < 12.0 °Brix, consumer acceptance was controlled by the interaction between TA and SSC rather than SSC alone. Our results showed only four genotypes with the mean value of TA > 0.9% and the mean value of SSC < 12.0 °Brix (3, 14, 58, 65).

### 2.2. Correlations between Agronomical and Basic Biochemical Traits

Significant correlations were found among pomological traits related to fruit quality. In the progeny, annual yield was positively correlated with fruit weight (*r* = 0.278, *P* ≤ 0.05), also a positive correlation was found for SSC and RI (*r* = 0.263, *P* ≤ 0.05). Firmness was significantly correlated with SSC (*r* = 0.367, *P* ≤ 0.01), pH (*r* = 0.385, *P* ≤ 0.01) and RI (*r* = 0.347, *P* ≤ 0.01). Similar low correlations were found for Cantín *et al.* when studied 1100 peach genotypes [17]. The positive correlation between firmness and SSC is important since the selection of genotypes with high SSC will aim first at higher firmness and second at lower susceptibility to mechanical damage during handling and packaging [26]. The ripening index showed a high positive correlation with pH (*r* = 0.930, *P* ≤ 0.01), and a negative correlation with titratable acidity (*r* = −0.315, *P* ≤ 0.01) indicating that in our progeny when most of the fruits are mature pH seems to increase and acidity to decrease. The pH showed a negative correlation with TA (*r* = −0.343, *P* ≤ 0.01) in this progeny, as other authors reported in different peach genotypes [10,16].

### 2.3. Antioxidant Compounds Content

The antioxidant compounds content in the “Venus” × “Big Top” progeny, showed a high variability among genotypes (Table 2). The ascorbic acid (AsA) content ranged from 2.1 to 7.2 mg of AsA/100 g of FW, with a mean value of 4.0 ± 0.1 mg of AsA/100 g of FW. The parents, “Venus” and “Big Top”, differed for vitamin C content, and as a consequence the progeny showed high segregation among genotypes. Our results indicate that peach is a good source of vitamin C and highlight the fact that ascorbic acid content is an important part of the overall evaluation of peach fruit quality. Values were in the same range as previously reported for vitamin C contents in peach flesh, namely 1–14 mg of AsA/100 g of FW [27]. Cantín *et al.* [10] reported that total ascorbic acid content in 218 peach genotypes from different progenies varied greatly from approximately 1 to 9 mg of AsA/100 g of FW, with a mean value of 3.7 mg of AsA/100 g of FW. Preliminary data obtained by these authors in this progeny but only tested during one year of study, showed lower values (2.6 mg of AsA/100 g of FW) when studying a short number of genotypes. Also Gil *et al.* [28] quantifying the total ascorbic acid contents of nectarine cultivars from California reported contents from 6 to 8 mg of AsA/100 g of FW in yellow-flesh nectarines and from 5 to 14 mg/100 g of FW in white-flesh nectarines.

Total phenolics ranged from 22.5 to 49.2 mg of gallic acid equivalent (GAE) per 100 g of FW. The amount of total phenolics in our progeny fell within the range reported in the literature for peach fruits, namely 14–77 mg GAE/100 g of FW. Tavarini *et al.* [27] reported similar total phenolics amounts (14–50 mg GAE/100 g of FW) in peach cultivars and other similar results were reported by Gil *et al.* [28] in yellow-flesh nectarines (18 to 54 mg GAE/100 g of FW).

Regarding flavonoids, it ranged from 5.9 to 33.8 mg catechin equivalent (CE) per 100 g of FW in our progeny with an average of 12.5 ± 0.6 mg of CE/100 g of FW. These results, revealed flavonoids content similar to that obtained by Cantín *et al.* [10] in peach and nectarine progenies ranging from 1.8 to 30.9 mg of CE/100 g of FW, with an average of 8.8 mg of CE/100 g of FW. It is remarkable that the “Venus” × “BigTop” progeny showed higher total phenolics and flavonoids content when compared with the parents. This fact could be very interesting in the peach genotype selection process, mainly selecting fruits rich in flavonoids, since the consumption of flavonoid-rich foods holds the potential to limit neurodegeneration preventing age-dependent loses in cognitive performance [29].

The anthocyanins content ranged from 1.2 to 9.5 mg cyanidin-3-glucoside equivalents (C3GE) per kg of FW, showing less variability among genotypes due to the similar flesh color of seedlings and lower concentrations compared to the study of Cantín *et al*. [10] who reported that in some progenies total anthocyanins greatly varied among genotypes (0.1–26.7 mg of C3GE/kg of FW) depending on the red pigmentation of the flesh.

The relative antioxidant capacity (RAC) ranged from 292.4 to 835.8 (μg Trolox Equivalents (TE) per g of FW) showing a high variability among genotypes (mean value was 462.2 ± 12.5 μg TE/g of FW). This could be explained by the fact that the antioxidant capacity of fruits varies in relation to the antioxidant molecules present in the different species but variations can also occur within the genotypes of a single species [28]. Cantín *et al.* [10] reported values of RAC (227.3 to 629.9 μg o f TE/g o f FW, with an average o f 4 0 5 μg o f T E/g o f FW) in the same range of these results even slightly lower. In general, antioxidant compounds content presented here were higher than those found for these authors when less genotypes of the same progeny were analyzed.

To evaluate the influence of the different environmental conditions on the fruit antioxidant compounds content, data related to 2007, 2008, 2009 and 2010 were separately evaluated (Table 3).

An interesting year-to-year and genotype-to-genotype variability differences in the antioxidant compounds were outlined. The vitamin C content showed higher values in 2009, whereas total phenolics, flavonoids and RAC showed comparable values among years. The anthocyanins content showed similar mean values in 2009 and 2010 but higher than those observed in the previous two years of the study, this could be due to the period of maturity of fruit and the harvest date which were more similar in those years. In 2008, the total phenolics, flavonoids content and the antioxidant capacity of the flesh fruit were notably higher than in the other years. These changes found in the antioxidant compounds content could be as a result of growing conditions, pre and postharvest conditions and genetic factors affecting the antioxidant compounds content. Harvesting date showed significant negative correlation with flavonoids (*r* = −0.331, *P* ≤ 0.01) and sucrose content (*r* = −0.310, *P* ≤ 0.01) indicating that harvesting time could present variability among years and consequently influence the antioxidant and total sugar content among genotypes.

The antioxidant content in the analyzed fruits should be attributed in part to the important role of the rootstock on fruit quality as Giorgi *et al*. reported [30]. Moreover, the environmental conditions to which the genotypes are subjected, and the annual climatic changes may partly explain the different accumulation patterns of antioxidant compounds. As a consequence, only the evaluation of several years of harvest may lead to an accurate assessment in the selection of new peach-rootstock combination [30,31].

**Figure 1:**
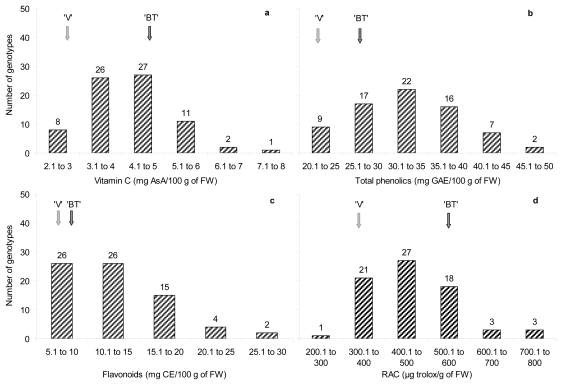
Segregation of (**a**) vitamin C, (**b**) total phenolics, (**c**) flavonoids, and (**d**) antioxidant capacity (RAC), in the “Venus” × “Big Top” progeny. Data are means (*n* = 42–75 genotypes) of four years of study (2007–2010). Arrows show the values for the parents “Venus” (‘V’) and “Big Top” (‘BT’).

All the antioxidant traits studied, except for flavonoids and anthocyanins (data not shown), showed a normal distribution (Figure 1) which is typical of quantitative characters.

Segregation was also observed when comparing the progeny with its parents, and some genotypes showed even higher values than “Venus” and “Big Top”. For vitamin C, at least ten genotypes (12, 27, 37, 38, 39, 40, 42, 43, 52 and 74) showed higher vitamin C contents. For total phenolics most of the progeny (5, 10, 18, 20, 23, 32, 35, 37, 40, 43, 44, 47, 61 and 74) showed higher contents compared with “Venus” and “Big Top”. The same thing occurred in the flavonoids content and these genotypes (10, 23, 28, 35, 37, 40, 43, 44, 47, 61 and 74) showing higher contents compared with the progenitors. For the relative antioxidant activity, seventeen genotypes (9, 10, 18, 20, 21, 23, 24, 27, 32, 35, 36, 37, 43, 44, 47, 61 and 74) showed values higher than the progenitors. In general, at least nine genotypes (18, 27, 32, 35, 37, 43, 44, 47 and 74) can be highlighted due to their higher contents of antioxidant compounds.

### 2.4. Total Sugar Content

The sucrose, glucose, fructose and sorbitol contents in the “Venus” × “Big Top” progeny were analyzed separately (Table 4), as they play an important role in peach flavor quality [32].

The studied population exhibited considerable phenotypic variation in sugar contents among genotypes. Mean values for all sugars were inside the interval values obtained for the parents, except for glucose that were higher, and these contents exhibited continuous variation, which is typical of quantitative or polygenic inheritance. Sucrose was the major sugar present in the evaluated genotypes, with 58.4 ± 1.2 g per kg FW, followed by fructose, glucose and sorbitol. The sorbitol content varied greatly among genotypes, ranging from 1.7 to 19.5 g per kg FW. Consequently, the percentage of sorbitol in the sugar composition was significantly different among genotypes, ranging from 1.1 to 8.7%. Colaric *et al.* [33] reported that sorbitol was the attribute most related to peach aroma and taste among carbohydrates and organic acids.

Wu *et al.* [34] reported that sucrose in peaches is dominant at maturity, followed by the reducing sugars (glucose and fructose) and then sorbitol. In our progeny the mean levels of glucose and fructose were quite similar (mean glucose/fructose ratio = 0.98) and about five times lower than the mean value for sucrose (mean sucrose/glucose ratio = 4.9). Some researchers reported glucose and fructose in comparable amounts [34]. However, a slight variation in glucose/fructose ratio (from 0.8 to 1.2) was detected in the studied seedlings. Identifying genotypes with low glucose/fructose ratio might be of particular interest, since the relative concentrations of these sugars influence sweetness, as fructose is 2.3 times and 1.7 times sweeter than glucose and sucrose, respectively [15]. In agreement, Robertson and Meredith [32] found that “high quality” peaches contained lower glucose/fructose ratios than “low-quality” peaches.

Total sugar content (the sum of sucrose, glucose, fructose and sorbitol) in peeled fruits ranged from 67.4 to 138.9 g per kg FW with an average of 89.7 ± 1.6 g per kg FW. Total sugar content is an important quality trait in fruit breeding programs, since it has been reported to be highly related to the aroma and taste of peaches and nectarines [33]. The “Venus” cultivar showed lower content than that observed by Colaric *et al.* [33]. Quilot *et al.* [21] reported that for total sugar content, variation among trees, among fruits of the same tree, and among years are not negligible in comparison with the variation among genotypes. Cantín *et al.* [14] studying 205 genotypes from different progenies reported that the average content of total sugars in the peeled fruit was 72.1 g per kg FW in peaches and 77.1 g per kg FW in nectarines.

**Figure 2:**
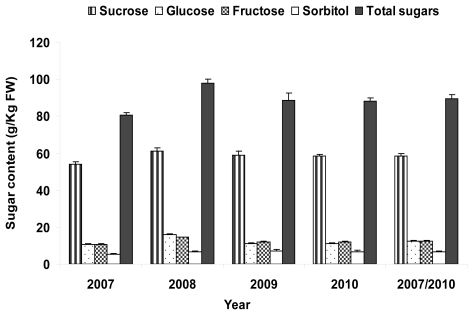
Annual amounts of sugar compounds (g per kg of FW) in the “Venus” × “Big Top” progeny. Data are means ± SE of four years of study (2007–2010) (*n* = 42–75 genotypes).

The annual variation for sugars compounds and total sugar content (Figure 2) showed small variation among years except for 2008. This year high values of sucrose, glucose and fructose were obtained leading to high total sugar content in agreement with the high SSC found (15.5 °Brix).

### 2.5. Correlations between Phytochemicals Traits

We found significant positive correlations (*P* ≤ 0.01) of relative antioxidant capacity *versus* total phenolics (Table 5), flavonoids, and vitamin C (*r* = 0.738, *r* = 0. 851 and *r* = 0.455, respectively), implying that they are important bioactive compounds for the antioxidant activity of peaches, in accordance with Cantín *et al.* [10]. The DPPH assay for the RAC determination explains the correlation found with total phenolics. The high positive correlation found between total phenolics and flavonoids content (*r* = 0.807, *P* ≤ 0.01) (Table 5), indicates that flavonoids are an important group of phenolic compounds in peaches and nectarines with high antioxidant activity. Moreover, total sugars showed positive significant correlations with total phenolics (*r* = 0.398), vitamin C (*r* = 0.350), and RAC (*r* = 0.384) at *P* ≤ 0.05. Pirie and Mullins [35] reported a good correlation in grapes between sugar content in berries and levels of phenolic substances, due to the role of sugars in the regulation of phenolic biosynthesis. Linear regression between RAC and total phenolics and flavonoids were also high (*r* = 0.738 and *r* = 0.851, respectively at *P* ≤ 0.01, data not shown). Similarly, Gil *et al.* [28] reported a strong correlation (*r* = 0.93–0.96) between total phenolics and antioxidant activities in fresh nectarine and peach fruits. It is well established that a strong and positive relationship exists between total phenolics and total anthocyanins content and RAC, suggesting that breeders can select for higher phenolics.

### 2.6. Principal Component Analysis for Agronomical and Biochemical Traits

**Figure 3:**
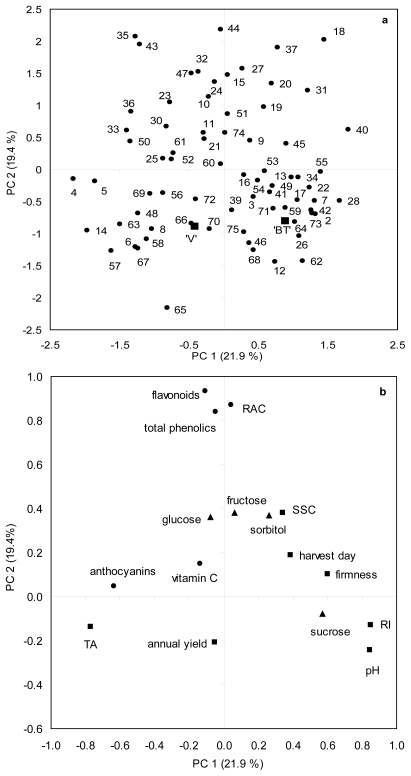
Principal component analysis of agronomical and biochemical traits in the “Venus” × “Big Top” progeny. Analysis was performed using mean data of four years of study (2007–2010). PC1/PC2 scores plot (a) explaining 41.3% of the total variance. Symbols: (■) parents “Venus” (‘V’) and “Big Top” (‘BT’), (●) progeny. PC1/PC2 loadings plot (b) generated from PCA analysis. Symbols: (■) agronomical and basic biochemical traits, (●) antioxidants, (▴) sugars.

A Principal Component Analysis (PCA) was performed on agronomical and biochemical data in the “Venus” × “Big Top” progeny (Figure 3). A four component model accounted for more than 70% of total variance, with the first two components, PC1 and PC2, explaining 21.9% and 19.4% of total variance, respectively. Progeny displayed a great variability (Figure 3a). PC1 discriminated between parental acid “Venus” and non-acid “Big Top”. An examination of PC1 loadings (Figure 3b) suggested that this separation was mainly due to basic biochemical traits (TA, pH and RI). Genotypes on the positive side of PC1 were in general less acid, showed higher firmness and accumulated more sugars and less anthocyanins than individuals on the negative side. An examination of PC2 loadings (Figure 3b) suggested that separation on this component was mainly due to antioxidant traits (flavonoids, total phenolics and RAC) and in a less extent to sugar compound accumulation (glucose, fructose and sorbitol). Analysis confirmed the higher contents in total phenolics, flavonoids and RAC for some genotypes (individuals on the positive side of PC2, especially 18, 27, 32, 35, 37, 43, 44 and 47) than progenitors. The PCA shows a close relationship between flavonoids, total phenolics and RAC as well as between RI, pH and firmness. The results obtained in this progeny were coherent and reflected the known correlations between bioactive and agronomical traits as described in others studies [14,17].

## 3. Experimental Section

### 3.1. Plant Material

The progeny assayed was a segregant F1 population of 75 seedlings obtained from a controlled cross, between *Prunus persica* cvs. “Venus” (female parent) and “Big Top” (male parent). “Venus” is a freestone, melting and yellow flesh nectarine cultivar, whereas “Big Top” is a clingstone, melting and yellow flesh nectarine cultivar. The segregant population is entirely melting flesh, either cling- or freestone. The resulting seedlings were budded on the same rootstock (GF 677) and established (one tree per genotype) in an experimental orchard at the Experimental Station of Aula Dei-CSIC (northern Spain, Zaragoza) in 2002. Trees were trained to the standard open vase system and planted at a spacing of 4 m × 2.5 m. Hand-thinning was carried out to reduce fruit load when required. Trees were grown under standard conditions of irrigation, fertilization and pest and disease control. Samples were harvested over four years (2007–2010).

### 3.2. Agronomical and Basic Fruit Quality Parameters

During the years 2007–2010, agronomic and fruit quality traits were measured individually in each seedling tree. Harvesting date and annual yield were evaluated in each independent seedling. Harvesting date ranged from first-July to mid-August, depending on the genotypes. Fruits were handpicked at commercial maturity and assessed by peel fruit color and flesh firmness. Fruits were considered ripe in the tree when their growth had stopped, exhibited orange-red ground color, began softening, and were easily detached. Yield (kg/tree) was measured and a representative fruit sample (20 fruits) was taken for fruit quality evaluations [10]. Fruit weight was also scored. Flesh firmness measurements were performed by a hand penetrometer with an 8 mm flat probe in two opposite sides of the fruit that had previously been peeled to remove the epidermis and data were expressed in Newtons. The SSC of the juice was measured with a temperature compensated refractometer (model ATC-1, Atago Co., Tokyo, Japan) and data are given as °Brix. The initial pH and titratable acidity (TA) were measured by automatic titration system with NaOH 0.1 N to pH 8.1 (862 Compact Titrosampler); data are given as g malic acid per 100 g FW, since this is the dominant organic acid in peach. The ripening index (RI) was calculated as the ratio between SSC and TA.

### 3.3. Phytochemical Extraction

For all analyses only fruit flesh was used, as it is usually consumed. Twenty representative fruits were peeled with a sharp knife, flesh was weighted, immediately frozen separately in liquid nitrogen, and stored at −20 °C until analysis. For vitamin C determination, samples at harvest were kept in 5 mL of 5% metaphosphoric acid for preservation of ascorbic acid, frozen in liquid nitrogen and stored at −20 °C until analysis. Samples were homogenized with a polytron in 5 mL 5% metaphosphoric acid and centrifuged at 20,000 g for 20 min at 4 °C, filtered with Miracloth and the supernatant was used for vitamin C analysis. For phenolic compounds, frozen fruit material (5 g) was homogenized in a polytron with 10 mL of extraction solution, consisting of 0.5 N HCl in methanol/Milli-Q water (80% v/v). The mixture was then centrifuged for 20 min at 4 °C and 20,000 g. Supernatant was recovered and the volume measured. This hydroalcoholic extract was used for total phenolics, flavonoids, anthocyanins and antioxidant capacity assays. For the determination of sugars, the frozen fruit material (5 g) was homogenized in a Polytron with 10 mL of extraction solution consisting of ethanol/Milli-Q water (80% v/v). The mixture was centrifuged at 20,000 g for 20 min at 4 °C. The supernatant was recovered and processed to be assayed by high-performance liquid chromatography (HPLC) as described by Cantín *et al.* [14] with some modifications.

### 3.4. Antioxidant Compounds Analysis

Vitamin C, total phenolics, flavonoids, anthocyanins and relative antioxidant capacity were evaluated with colorimetric methods and measured using a spectrophotometer (Beckman Coulter DU 800) as described by Cantín *et al*. [10] and methods therein. In order to avoid interferences, other analysis could be recomended for specific determinations of anthocyanins, flavonoids and total phenolics [36,37]. Standard calibration curves were daily prepared. For vitamin C determinations, absorbance was measured at 525 nm and the amount of vitamin C was expressed as mg of ascorbic acid (AsA) per 100 g fresh weight (FW). For total phenolics content, the colorimetric method based on the chemical reduction of the Folin-Ciocalteau reagent was used. Absorbance was measured at 725 nm and the content was expressed in milligrams of gallic acid (3,4,5-Trihydroxy-benzoic acid) equivalents (GAE) per 100 g of FW. Total flavonoids content was determined measuring absorbance at 510 nm and the results were expressed as milligrams of catechin equivalents (CE) per 100 g of FW. The total anthocyanins content was evaluated measuring in the hydroalcoholic extract the absorbance at 535 and 700 nm. The anthocyanins concentration was calculated using the molar extinction absorptivity coefficient ɛ = 25,965/cm M and was expressed in mg of cyanidin 3-glucoside equivalents (C3GE) per kg of FW. The relative antioxidant capacity (RAC) was determined using the 1,1-diphenyl-2-picrylhydrazyl (DPPH). The absorbance was measured after 10 min of reaction at 515 nm and RAC was expressed as μg of Trolox equivalents per g of FW.

### 3.5. Total Sugars Analysis

To estimate the variation in sugar profile among genotypes, sugar composition and quantification were measured as described by Cantín *et al.* [14]. For the analysis, 250 μL of the homogenized extract was incubated at 80 °C for 20 min in 200 μL of 800 mL/L ethanol, with 5 g/L manitol added as an internal standard. Samples were purified using ion exchange resins (Bio-Rad Barcelona, Spain) [38]. Twenty μL was injected into the HPLC system (Aminex HPX-87C column, 300 mm × 7.8 mm; Bio-Rad, Barcelona, Spain) with a refractive index detector (Waters 2410). The solvent was deionized water running at a flow rate of 0.6 mL per min at 85 °C. Sugar quantification was performed with Millenium 3.2 software (Waters) using standards of analytical grade (Panreac Quimica SA, Barcelona, Spain). Sugar concentrations were expressed as g per kg FW.

### 3.6. Statistical Analysis

All traits were measured or scored for each genotype separately over the four year period, and means of four years were calculated. All statistical analyses were performed using SPSS 19.0 (SPSS Inc., Chicago, IL). To obtain basic statistics for the entire plant material studied, minimum and maximum values, mean and mean standard error (SE), were calculated for each trait. Data for each genotype were averaged, and mean values were used as estimated genotypic values. Finally, correlations were calculated with raw data of the four years, according to Pearson’s test at *P* ≤ 0.01. Principal component analysis (PCA) was applied on the antioxidant compounds and basic agronomical traits in the studied population as an attempt to identify superior genotypes based on their antioxidant compound contents. The component matrix was evaluated and orthogonal factors were rotated using variance maximizing (Varimax).

## 4. Conclusions

To summarize, the progeny showed a great phenotypic variance for all the pomological and antioxidant studied traits. High variability was found in yield, fruit weight, firmness, SSC, TA, ascorbic acid, total phenolics, flavonoids, anthocyanins, antioxidant capacity, and total sugars which indicate that there is an important genetic potential to develop new nectarine cultivars with high fruit quality. On the other hand, the significant correlations found between some agronomical and quality attributes could be of interest for quality oriented fruit breeding programs. The study also emphasizes the usefulness of PCA in evaluating the fruit quality of new breeding releases and studying relationships among pomological traits.

Our results lead us to the conclusion that the antioxidant capacity of peach is characterized by huge levels of variations, much explained by the genotype, but harvest conditions and season may also be significant factors. Most of the progeny showed higher total phenolics and flavonoids content than parents. This fact could be of importance for selection of specific traits in the progeny. The phenotypic variation found in all studied traits will allow selecting superior genotypes with higher antioxidant content than the existing commercial varieties and this will naturally be beneficial for health.

## Figures and Tables

**Table 1:** Agronomical and basic fruit quality traits in the “Venus” × “Big Top” population. For progenitors data are means ± SE of two years (2009–2010). For the progeny (*n* = 75 genotypes), data are means ± SE of four years of study (2007–2010).

Progenitors			Progeny a		
	
Traits	“Venus”	“Big Top”	Min	Max	Mean ± SE
Fruit weight	178.0 ± 58.3	204.0 ± 39.3	109.2	261.8	190.2 ± 3.8
Firmness	36.1 ± 0.1	49.2 ± 6.9	24.2	50.7	39.2 ± 0.6
SSC	13.9 ± 0.1	14.4 ± 0.1	11.2	17.5	13.9 ± 0.2
pH	3.4 ± 0.1	3.9 ± 0.1	3.2	4.0	3.6 ± 0.1
TA	0.7 ± 0.1	0.4 ± 0.1	0.5	1.1	0.7 ± 0.1
RI	20.3 ± 0.3	35.2 ± 0.3	13.8	35.8	23.8 ± 0.7

a Data from 2007 were partially presented in Cantín *et al.* 2010 [17]. Units and abbreviations: Fruit weight (g); Firmness (N); N = Newtons; SSC = Soluble solids content (°Brix); TA = Titratable acidity (g malic acid/100 g FW); RI = Ripening index (SSC/TA).

**Table 2:** Content of antioxidant compounds in the “Venus” × “Big Top” population. For progenitors data are means ± SE of two years (2009–2010). For the progeny (*n* = 42–75 genotypes), data are means ± SE of four years of study (2007–2010).

Progenitors			Progeny		

Compounds	“Venus”	“Big Top”	Min	Max	Mean ± SE
Vitamin C	3.0 ± 0.7	4.9 ± 0.7	2.1	7.2	4.0 ± 0.1
Total phenolics	22.1 ± 8.0	26.4 ± 9.8	22.5	49.2	32.6 ± 0.7
Flavonoids	7.6 ± 3.8	7.8 ± 4.6	5.9	33.8	12.5 ± 0.6
Anthocyanins	2.1 ± 0.1	5.9 ± 2.2	1.2	9.5	3.2 ± 0.2
RAC	386.1 ± 18.5	521.4 ± 47.4	292.4	835.8	464.2 ± 12.5

Units: Vitamin C (mg AsA/100 g of FW); Total phenolics (mg GAE/100 g of FW); Flavonoids (mg CE/100 g of FW); Anthocyanins (mg C3GE/kg of FW); RAC; Relative Antioxidant Capacity (μg Trolox Equivalents/g of FW). Abbreviations: AsA = Ascorbic acid; C3GE = Cyanidin-3-glucoside equivalents; CE = Catechin equivalents; GAE = Gallic acid equivalents.

**Table 3:** Annual amounts of antioxidant compounds in the “Venus” × “Big Top” progeny. Data are means ± SE of four years of study (2007–2010) (*n* = 42–75 genotypes).

Compounds	2007	2008	2009	2010	Mean ± SE
Vitamin C	2.8 ± 0.1	3.9 ± 0.2	6.3 ± 0.2	3.2 ± 0.2	4.0 ± 0.1
Total phenolics	36.9 ± 1.7	44.2 ± 0.7	21.2 ± 0.6	23.3 ± 0.8	32.6 ± 0.7
Flavonoids	12.9 ± 1.0	21.7 ± 1.2	6.5 ± 0.4	8.1 ± 0.6	12.5 ± 0.6
Anthocyanins	2.2 ± 0.2	1.7 ± 0.1	4.0 ± 0.5	4.6 ± 0.4	3.2 ± 0.2
RAC	380.6 ± 14	617.0 ± 23	322.6 ± 11	444.8 ± 10	464.2 ± 12.5

Units: Vitamin C (mg AsA/100 g of FW); Total phenolics (mg GAE/100 g of FW); Flavonoids (mg CE/100 g of FW); Anthocyanins (mg C3GE/kg of FW); RAC; Relative Antioxidant Capacity (μg Trolox Equivalents/g of FW). Abbreviations: AsA = Ascorbic acid; C3GE = Cyanidin-3- glucoside equivalents; CE = Catechin equivalents; GAE = Gallic acid equivalents.

**Table 4:** Sugar content (g per kg FW) in the “Venus” × “Big Top” population. For progenitors data are means ± SE of two years (2009–2010). For the progeny (*n* = 42–75 genotypes), data are means ± SE of four years of study (2007–2010).

Progenitors			Progeny		

Sugar content	“Venus”	“Big Top”	Min	Max	Mean ± SE
Sucrose	41.0 ± 5.7	85.1 ± 17.0	40.7	102.3	58.4 ± 1.2
Glucose	10.0 ± 0.4	8.9 ± 0.8	8.3	23.4	12.2 ± 0.3
Fructose	13.4 ± 0.5	10.9 ± 1.7	8.9	19.1	12.4 ± 0.2
Sorbitol	8.6 ± 3.8	6.5 ± 1.7	1.7	19.5	6.6 ± 0.5
Sucrose/glucose	4.0 ± 0.4	9.5 ± 2.1	3.2	7.6	4.9 ± 0.1
Glucose/fructose	0.7 ± 0.1	0.8 ± 0.1	0.8	1.2	0.9 ± 0.1
% Sorbitol	5.6 ± 1.9	2.9 ± 0.4	1.1	8.7	3.5 ± 0.2
Total sugars	73.0 ± 9.6	111.5 ± 14.1	67.4	138.9	89.7 ± 1.6

**Table 5:** Correlation coefficients between some phytochemical traits in the “Venus” × “Big Top” progeny.

Traits	Flavonoids	Total phenolics	RAC	SSC	Total Sugars
Vitamin C	0.420 **	0.374 **	0.455 **	0.579 **	0.350 **
Flavonoids		0.807 **	0.851 **	0.482 **	ns
Total phenolics			0.738 **	0.524 **	0.398 **
RAC				0.597 **	0.384 **

** *P* ≤ 0.01; ns, not significant. Abbreviations: RAC; Relative Antioxidant Capacity.
